# Proteomic Analysis of Glioma Chemoresistance 

**DOI:** 10.2174/157015912799362733

**Published:** 2012-03

**Authors:** Kyoungho Suk

**Affiliations:** Department of Pharmacology, Brain Science and Engineering Institute, Kyungpook National University School of Medicine, Daegu, Korea

**Keywords:** Glioma, drug, proteome, chemotherapy, resistance, central nervous system, biomarker, omics, systems biology.

## Abstract

Malignant glioma is the most common and destructive form of primary brain tumor. Along with surgery and radiation, chemotherapy remains as the major treatment modality. The emergence of drug resistance, however, often leads to a therapeutic failure in the treatment of glioma, precluding long-term survival of the patients. A proteomic approach has recently been adapted for the mechanistic analysis of glioma drug resistance. The proteomic analysis of drug-resistant glioma led to the discovery of novel biomarkers that can be used for the prognosis of glioma as well as for monitoring the drug response or resistance of glioma. These proteomics-based biomarkers can also be a druggable target that one can exploit for successful glioma chemotherapy. In this review, recent reports on proteomic analysis of glioma from the perspective of chemoresistance are discussed with a focus on the proteome profiles of glioma cells that are resistant to the alkylating agent, 1, 3-bis (2-chloroethyl)-1-nitrosourea (BCNU), as a prime example. Among numerous proteins that were up- or down-regulated in drug-resistant glioma cells, lipocalin 2 (LCN2) and integrin β3 (ITGB3) were identified as key proteins that determine the survival and death of glioma cells. LCN2, ITGB3, and other proteins identified by proteomic analysis could be utilized to overcome glioma chemoresistance.

## INTRODUCTION

Glioma is a type of tumor that arises from glial cells in the central nervous system (CNS) [1]. Gliomas can be classified by cell type and grade. Astrocytomas, oligodendrogliomas, and ependymomas are derived from astrocytes, oligodendrocytes, and ependymal cells, respectively. According to the grade, gliomas are categorized into low-grade glioma (grade 2) or high-grade anaplastic or malignant glioma (grade 3-4). Glioblastoma multiforme (GBM) (grade 4) is the most prevalent and aggressive form of glioma in the brain. Malignant glioma and GBM are often resistant to treatment and have a poor prognosis. Standard therapies against malignant gliomas include surgery, radiation, and chemotherapy, which are often used in a combinatorial approach [2-4]. Currently, temozolomide is the drug of choice that is most commonly used in glioma chemotherapy because the drug crosses the blood-brain barrier and effectively improves clinical outcomes when used alone or in combination with radiotherapy [5, 6]. Malignant glioma is, however, rarely curable. Chemoresistance of glioma is one of the major problems in glioma therapy [7, 8]. In an attempt to gain biological insights into the molecular mechanism(s) of glioma chemoresistance, omics approaches have been recently employed. Transcriptomics-, proteomics-, and metabolomics-based studies generated large-scale data, which provided an integrative view of the biological processes. These omics technologies along with systems biology approach will be increasingly used to understand the molecular and cellular mechanisms underlying glioma drug resistance. Knowledge obtained at the systems level will then be translated to overcome chemoresistance in glioma patients. Here, recent proteomics-based approaches toward this goal will be briefly reviewed, and pivotal components of the decision-making process of glioma cell death/survival and chemoresistance will be discussed as well.

## DRUG RESISTANCE OF GLIOMA 

Chemotherapy remains as the mainstream treatment modality for malignant glioma [9]. After surgically removing as much of the tumor as possible, any remaining part of the tumor is treated with radiotherapy or chemotherapy. A common type of combination chemotherapy is composed of procarbazine, lomustine, and vincristine. The National Institute for Health and Clinical Excellence has also recommended temozolomide capsules and carmustine implants as a possible treatment for newly diagnosed GBM. Temozolomide and carmustine are alkylating agents, which work by damaging the DNA of cancer cells and stopping their proliferation. Carmustine is also called 1, 3-bis (2-chloroethyl)-1-nitrosourea (BCNU). These alkylating agents are the most commonly used therapeutic drugs against malignant glioma. Unfortunately, however, the drug treatment often fails to achieve long-term survival of glioma patients due to the emergence of drug resistance. It has been previously suggested that an increased expression of multidrug resistance (MDR) genes, DNA repair activity such as O6-methylguanine-DNA methyltransferase (MGMT), and detoxification activity such as glutathione S-transferase pi 1 (GSTP1) may be involved in causing chemoresistance to alkylating agents in glioma patients [7]. Studies also suggested the involvement of cancer stem cells, major-vault protein (MVP), anti-apoptotic protein BCL2, oncogenes, tumor suppressor genes, and protein kinase C, GRP78/BiP, in glioma chemoresistance [8, 10]. Although chemoresistance is the major therapeutic challenge in glioma, the precise molecular basis of chemoresistance remains largely unknown. 

## PROTEOMIC ANALYSIS OF GLIOMA

Heterogeneous biological features of glioma can be described by the global gene expression profile. Numerous studies have been done to compare the transcriptome profile of high and low-grade glioma tissues [11-13]. More recently, a proteome profile has also been compared between normal brain tissue and different grades of glioma tissue. Recent reviews by Whittle *et al*. [14] and Niclou *et al*. [15] nicely summarized the current status of glioma proteomics and its clinical applications. The glioma proteome has been previously analyzed using patient samples (glioma tissue or body fluid such as serum), cultured glioma cell lines, or animal models in an effort to enhance our understanding of glioma biology as well as to search for protein biomarkers that contribute to a better diagnosis and prognosis of glioma and to a better evaluation of drug responses to glioma [16]. In order to gain insights into the molecular characteristics of glioma, differential protein expression patterns between normal and glioma tissues have been analyzed by quantitative proteomic techniques such as two-dimensional gel electrophoresis (2DE), matrix-assisted laser desorption and ionization time of flight (MALDI–TOF), and liquid chromatography and tandem mass spectrometry (LC-MS/MS). In a recent study by Iwadate *et al*. [17], a proteomics-based discriminant analysis identified a set of 37 proteins differentially expressed in glioma versus the control, which were mostly categorized as signal transduction proteins. The authors indicated that these proteins could be used to predict the biological aggressiveness of glioma. Schwartz *et al*. did MALDI mass spectrometric analysis of glioma tissue samples to obtain a glioma-specific protein profile [18]. Based on the proteomic profile, they were able to predict tumor malignancy and patient survival. Although proteomics-based approaches in glioma research led to the identification of altered protein expressions, lack of consistency in the data emerged as a principal hurdle in establishing the biological significance of the laboratory findings. A systematic review by Deighton *et al*. of multiple independent proteomic analyses of glioma has demonstrated alterations in the abundance of 99 different proteins including PHB, Hsp20, serum albumin, epidermal growth factor receptor (EGFR), EA-15, RhoGDI, APOA1, GFAP, HSP70, and PDIA3 [19]. The authors emphasized the importance of network analysis: for example, protein-protein interaction analysis placed TP53 and RB1 at the core of the network for glioblastoma. That review provided an overview of the glioma proteome literature citing 10 published, peer-reviewed articles. In an attempt to find new potential diagnostic, prognostic, and predictive biomarkers for glioma, extensive studies have been done or are in progress. Some of the most promising biomarkers to date include loss of chromosomes 1p/19q in oligodendroglioma and expression of MGMT or EGFR status in glioblastoma. Other promising biomarkers include glial fibrillary acidic protein (GFAP), galectins, Kir potassium channel proteins, angiogenesis, and apoptosis pathway markers [20]. Fifty human brain glioma tissues with different grades (non-tumor and grades 1-4) were subjected to differential gel electrophoresis (DIGE) technology coupled with MALDI and LC-tandem MS [21]. Among 91 unique proteins identified, Alb protein, peroxiredoxin 4, and SH3 domain-binding glutamic acid-rich-like protein 3 were increased in GBM compared with non-tumor tissues. Aldolase C fructose-biphosphate, creatine kinase, B chain dihydrolipoyl dehydrogenase, enolase 2, fumarate hydratase, HSP60, lactoylglutathione lyase, leucine aminopeptidase, Mu-crystallin homolog, NADH-UO 24, neurofilament triplet L protein, septin 2, stathmin, and vacuolar ATP synthase subunit E were decreased in GBM versus non-tumor tissues.

**Fig. (1):**
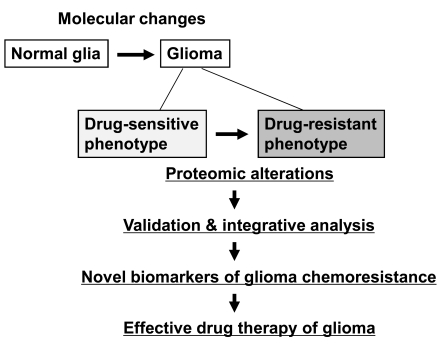
**Flowchart depicting the proteomic analysis of glioma chemoresistance.** Phenotypic changes of normal glial cells to glioma and its drug sensitivity involve a variety of molecular and proteomic alterations. Validation and integrative analysis of these proteomic alterations leads to the discovery of protein biomarkers that reflect the drug-resistant phenotype of glioma. The proteomics-based biomarkers will ultimately aid in successful and effective pharmacotherapy of glioma evading chemoresistance.

Glioma chemoresistance has also been a subject of proteomic investigation (Fig. **1**). Iwadate *et al*. identified a set of 41 proteins that affected chemosensitivity of human glioma to anticancer drugs [22]. They used 2DE analysis of 93 human glioma samples to find a correlation between the sensitivity to 10 different anticancer agents and the proteome profile of glioma. The study identified signal transduction proteins such as G proteins as potential predictive markers of chemosensitivity in human glioma. Okamoto *et al*. compared the proteome of oligodendrogliomas with different chemosensitivities to identify 7 candidate proteins that were over-expressed in chemoresistant oligodendroglioma [23]. Two of these proteins were glyoxalase I and Rho GDP dissociation inhibitor, which have previously been shown to enhance chemoresistance in other tumors. More recently, Rostomily *et al*. also performed quantitative proteomic analysis on oligodendroglioma with or without 1p/19q deletion [24]. Using microcapillary LC-MS along with a quantitative technique called isotope-coded affinity tags, they identified 163 non-redundant proteins with significant changes in relative abundance between the two different patient samples. Subsequent bioinformatic analyses of the differentially regulated proteins indicated the potentially important role of pro-invasive extracellular matrix protein BCAN in glioma malignancy. Glioma response to the chemotherapeutic agent platinum compounds was evaluated by proteomic profiling [25]. A two-dimensional chromatography system was used to search for protein biomarkers of drug response in glioma. Another similar study defined the proteomic profile of glioblastoma cells exposed to terpyridineplatinum(II) complexes (TPCs), a potent and specific inhibitor of human selenoprotein thioredoxin reductase (TrxR) [26]. TPC treatment resulted in a spectrum of cellular events in glioblastoma including upregulation of TrxR expression, activation of p53 and its downstream molecules, and endoplasmic reticulum stress.

Analysis of protein pattern differences was also done to compare glioma cell lines and GBM [27]. Proteomic comparison among 4 glioma cell lines (U87, U118, U251, and A172 cells) and microdissected GBM tissues using 2DE followed by LC-MS/MS identified 17 proteins that were significantly up- or down-regulated in cultured cell lines compared with GBM tissues. Transcription factors, tumor suppressor genes, cytoskeletal proteins, and cellular metabolic proteins were included in the list of proteins identified. A similar study by Zhang *et al*. identified differentially expressed proteins in human glioblastoma cell lines versus tumors [28]. Proteomic profiling in fetal human astrocytes and human glioblastoma cell lines U87MG and U87MG expressing type III EGFR deletion was followed by Western blot, ELISA, or RT-PCR in cell extracts and in tumor tissues to discover potential biomarkers like Hsp27, major vault protein, tissue transglutaminase, and cystatin B. These results point to the advantages and limitations of cell culture studies. The U87MG glioblastoma cell line expressing mutant EGFR was previously established by retroviral transfer of EGFR gene with an in-frame deletion of 801 bp of the coding sequence of the extracellular domain [29]. Furuta *et al*. applied the proteomic approach to identify subtypes of GBM [30]. Primary or secondary tumors of GBM were successfully distinguished by specific proteomic patterns. Different grades of astrocytomas were also differentiated according to 2DE-based proteomic patterns together with immunohistochemical validation [31]. Proteomics-based biomarkers can be utilized to investigate potential associations with specific biomarkers and drug resistance of glioma. Drug response profiles for 478 biopsy specimens from patients with different stages of glioma were determined [32]. Resistance to anti-cancer agents such as BCNU, cisplatin, dacarbazine, paclitaxel, vincristine, and irinotecan was associated with drug resistance biomarkers such as multidrug resistance gene-1, GSTP1, MGMT, and mutant p53. Proteomics has also been used to study chemosensitivity or chemoresistance for several other types of cancers including neuroblastoma [33], melanoma [34], pancreatic carcinoma [35], breast cancer [36], and gastric cancer [37].

## IDENTIFICATION OF KEY PROTEINS INVOLVED IN GLIOMA CHEMORESISTANCE: PROTEOME PROFILE OF DRUG-RESISTANT C6 GLIOMA CELLS AS A MODEL

**Fig. (2):**
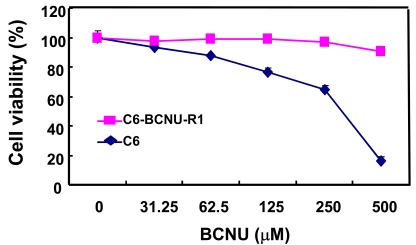
**Comparison of drug sensitivity between C6 glioma cells and C6-BCNU-R1 variant cells.** Parental as well as variant glioma cells were treated with an increasing concentration of BCNU for 24 hr, and cell viability was assessed by MTT assay.

**Fig. (3):**
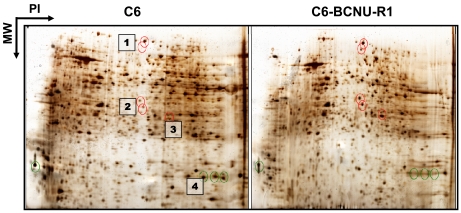
**2DE analysis of glioma chemoresistance.** Proteome profiles of drug-sensitive versus resistant glioma cells were compared by 2DE analysis. Several representative protein spots showing differential expression were subjected to MALDI-TOF identification. Identity of spots 1-4 is shown in Table **1**.

**Fig. (4):**
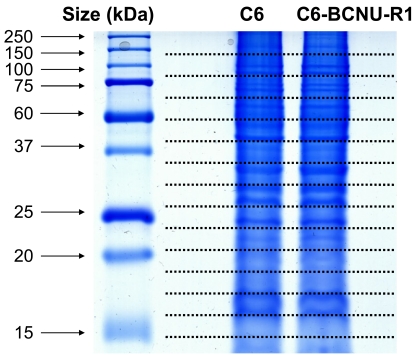
**Separation of proteins isolated from drug-sensitive C6 glioma cells and their drug-resistant variant C6-BCNU-R1 cells.** Total proteins were separated by SDS-PAGE and the gel was cut into 15 slices for LC-MS/MS analysis. C6, drug-sensitive; C6-BCNU-R1, drug-resistant.

In order to investigate the proteome-based mechanism of glioma chemoresistance, a variant of C6 rat glioma cells that is resistant to chemotherapeutic agents was established, and its proteome profile was compared with that of drug-sensitive parental cells [38]. C6 glioma cells were cultured *in vitro* in the presence of low concentrations of BCNU for a long period of time to induce phenotypic changes (Fig. **2**). A variant of C6 cells with drug-resistant phenotype (C6-BCNU-R1) was established by cell cloning, whose proteome profile was then determined by 2DE-MALDI-TOF or LC-MS/MS [39]. The 2DE followed by mass spectrometric analysis identified several protein spots that showed differential expression between C6 and C6-BCNU-R1 cells (Fig. **3** and Table **1**). Expression of protein disulfide-isomerase A3 precursor and proteasome subunit alpha type 6 was increased, while peptidyl-prolyl cis-trans isomerase A (cyclophilin A) expression was decreased in the drug-resistant glioma cells (C6-BCNU-R1). Previously, cyclophilin A or B was associated with cancer cell resistance to apoptotic cell death [40, 41]. Proteome profiles of the drug-sensitive and resistant glioma cells were also compared by LC-MS/MS. Glioma cell proteins were first separated by one-dimensional SDS-PAGE electrophoresis, and whole gel lanes were cut into 15 slices of equal size. The excised gel slices were subjected to LC-MS/MS analysis (Fig. **4**). More than 9 proteins were identified to be up- or down-regulated in the chemoresistant glioma cells (Tables **2**,**3**). Since glioma cells secrete proteins that influence glioma cell death/survival and malignancy in an autocrine or paracrine manner, secretomic analysis was also done. Comparison of secreted proteomes between the drug-sensitive and resistant glioma cells indicated that 20 proteins showed differences in abundance (Tables **4**,**5**). Among the proteins whose abundance was up-regulated, cathepsin L precursor [42], nexin (serpine2) [43], and extracellular superoxide dismutase [Cu-Zn] precursor [44, 45] were previously associated with glioma cell migration and death/survival. These proteins identified by proteomic analysis may provide an important clue in understanding glioma chemoresistance. Nevertheless, it should be noted that proteomic profiling may have potential caveats. Both gel-based and non-gel-based proteomic methods have unique advantages and limitations. For example, the SELDI method has been previously used to obtain a quantitative profile of the cellular proteome; however, it is unable to identify individual proteins. Quantitative gel-free MS-based platforms are increasingly used with a recent progress in MS instrumentation. New approaches for the improvement of current MS-based profiling are also emerging in combination with powerful fractionation strategies and antibody-based assays [46]. These new methodologies will improve the identification of drug resistance markers in glioma.

**Fig. (5):**
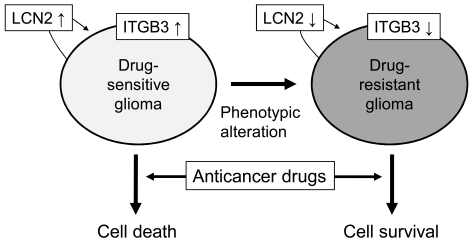
**Central role of LCN2 and ITGB3 in the phenotypic change of glioma chemosensitivity.** Downregulation of the secreted protein LCN2 and the membrane protein ITGB3 renders glioma cells resistant to anticancer drugs.

Among numerous proteins identified by the proteomic comparison of drug-sensitive and resistant glioma cells, lipocalin 2 (LCN2) [38] and integrin β3 (ITGB3) [39] played a central role in regulating glioma chemosensitivity (Fig. **5**). LCN2 is a member of the lipocalin family, which binds or transports lipids and other hydrophobic molecules [47]. LCN2 is also called neutrophil gelatinase-associated lipocalin (NGAL) or 24p3. LCN2 is involved in diverse biological processes such as iron delivery, the innate immune response to bacterial infection, cell death/survival, and many types of human cancers [48-51]. Our previous study showed that LCN2 gene expression was significantly down-regulated in the phenotypically selected BCNU-resistant C6 glioma cells [38]. Further studies such as recombinant LCN2 protein treatment, forced expression, or knockdown of LCN2 gene expression in glioma cells revealed that LCN2 downregulation played a key role in the BCNU resistance of glioma cells. LCN2 enhanced BCNU-induced Akt dephosphorylation providing a molecular basis for the apoptosis-sensitizing effects of LCN2. These results suggest that LCN2 protein may be involved in glioma drug resistance. ITGB3 was another protein whose expression was significantly down-regulated in the BCNU-resistant C6 glioma cells [39]. Integrins are integral membrane proteins that play key roles in glioma activities. A combination of 18 different α subunits and 8 different β subunits make up twenty-four distinct integrins [52-53]. ITGB3 is a component of integrin αvβ3, which has multifaceted functions in tumor cells including cell growth, adhesion, migration [54-55], tumor progression/invasion, growth factor response [56], and angiogenesis [57]. It has been previously shown that a significant downregulation of ITGB3 was associated with BCNU-resistance in C6 glioma cells [39]. There was a positive correlation between the ITGB3 expression level and the chemosensitivity to anticancer drugs in human glioma cells. Moreover, nitric oxide (NO) enhanced anticancer drug-induced glioma cell death by increasing ITGB3 expression. Pharmacological and biochemical studies indicated pro-apoptotic functions of ITGB3 in glioma cells, which may be mediated by Erk activation and the unligated integrin-mediated cell death (IMD) pathway. Taken collectively, previous studies provide the evidence for the pro-apoptotic role of LCN2 and ITGB3 in glioma cells. LCN2 and ITGB3 could be exploited to develop a new therapeutic approach to sensitizing glioma to anticancer drugs. Expression of LCN2, ITGB3, and other specific proteins should also be evaluated in glioma tissues of the patients in future studies.

## CONCLUSIONS

Omics or systems biology approaches have been used to study chemoresistance of glioma. With a recent interest in moving toward an integrative, rather than reductionist, approach to glioma biology in the post-genomic era, proteomic pattern comparisons between normal and glioma tissues of different grades led to the discovery of numerous potential biomarkers that could be translated into diagnosis or prognosis in the clinical field. The drug response or resistance of glioma has also been an area of intensive investigation. Proteomic profiling of glioma chemoresistance identified multiple candidate proteins that may be responsible for glioma acquisition of drug resistant phenotypes. LCN2, ITGB3, and other proteins identified by proteomic analysis of glioma chemoresistance may help overcome drug resistance of glioma and improve clinical outcomes of patients. 

Omics approaches, proteomics in particular, offer a promising outlook that would revolutionize the discovery of novel biomarkers for monitoring drug response. Inconsistency in large-scale data of proteomic research in the literature is, however, a major problem that needs to be solved. Focused and thorough validation, use of multiple protein biomarkers rather than single ones, and combination with imaging markers or other means should be highlighted in the future to strengthen the power of biochemical markers in predicting glioma prognosis and drug response. Pathway or network-oriented interpretation of multiple candidate biomarkers would also help gain an integrative view of glioma chemoresistance. Systems biology-based approaches will be increasingly used with the advancement of proteomic technology in the study of glioma chemoresistance to gain wider perspectives on the pathophysiology of brain tumors.

## Figures and Tables

**Table 1.** List of Proteins Identified by 2DE and MALDI-TOF

Spot No.	Protein Identity	Symbol	MW (kDa)
1	Aldehyde dehydrogenase, mitochondrial precursor	Aldh2	56
2	Protein disulfide-isomerase A3 precursor	Pdia3/ERP57	57
3	Proteasome subunit alpha type 6	Psma6	27
4	Peptidyl-prolyl cis-trans isomerase A	Ppia/CYPA	17

After separation of the proteins by 2DE from the glioma cells (Fig. **3**), spots were cut and subjected to MALDI-TOF identification.

**Table 2.** Partial list of Proteins Up-Regulated in Drug-Resistant Glioma Cells

Gene Symbol	Gene Name	IPI Number	Peptide Fold Increase
Nqo1	NAD(P)H dehydrogenase [quinone] 1	IPI00231595	13.7
Dpysl3	Isoform 2 of Dihydropyrimidinase-related protein	IPI00203250	7.4
Rtn4	Isoform 1 of Reticulon-4	IPI00231765	8.5
Eef1a1	Elongation factor 1-alpha 1	IPI00195372	77
Oat	Ornithine aminotransferase, mitochondrial precursor	IPI00192793	4

**Table 3.** Partial list of Proteins Down-Regulated in Drug-Resistant Glioma Cells

Gene Symbol	Gene Name	IPI Number	Peptide Fold Decrease
Cdc2a	Cell division control protein 2 homolog	IPI00190390	11.3
Lmna	Lamin-A	IPI00201060	11.4
Cald1	Non-muscle caldesmon	IPI00208118	8.7
Nudc	Nuclear migration protein nudC	IPI00210009	5.2

**Table 4.** Partial List of Secreted Proteins Up-Regulated in Drug-Resistant Glioma Cells

Gene Symbol	Gene Name	IPI Number	Peptide Ratio (C6/C6-BCNU-R1)
Ctsl	Cathepsin L precursor	IPI00326070	15/22
Emilin1	Predicted similar to elastin microfibril interfacer 1	IPI00199867	4/30
Fn1	Isoform 1 of fibronectin precursor	IPI00200757	314/515
Loxl3	Predicted similar to lysyl oxidase-like 3	IPI00365102	2/29
Mfge8	Milk fat globule-EGF factor 8 isoform 1 precursor	IPI00559274	31/60
Mmp10	Stromelysin-2 precursor	IPI00204362	0/10
Mmp3	Stromelysin-1 precursor	IPI00324928	21/40
Nrp2	Neuropilin 2	IPI00562238	4/12
Tgfbi	Transforming growth factor, beta induced	IPI00188622	14/19
Serpine2	Similar to glia-derived nexin precursor	IPI00203479	33/56
C1s	Complement component 1, s subcomponent	IPI00199519	15/20
Clstn1	Calsyntenin 1	IPI00765417	3/11
Sod3	Extracellular superoxide dismutase [Cu-Zn] precursor	IPI00200507	14/18

**Table 5.** Partial List of Secreted Proteins Down-Regulated in Drug-Resistant Glioma Cells

Gene Symbol	Gene Name	IPI Number	Peptide Ratio (C6/C6-BCNU-R1)
Bgn	Biglycan precursor	IPI00191090	35/28
Hemiferrin	Hemiferrin	IPI00213667	54/41
Msn	Moesin	IPI00778167	22/5
Pcolce	Procollagen C-endopeptidase enhancer 1 precursor	IPI00194566	25/17
Thbs2	Thrombospondin-2 precursor	IPI00734663	19/0
Thbs1	Thrombospondin 1	IPI00422076	3/0
Timp2	Putative uncharacterized protein of 24 kDa	IPI00777750	17/13
Spp1	Osteopontin precursor	IPI00327895	26/5
Serpinf1	Alpha-2 antiplasmin	IPI00199670	24/17
Sema3a	Semaphorin-3A precursor	IPI00210066	9/5
Tenascin	Tenascin	IPI00208020	62/48
Col12a1	Similar to Collagen alpha-1(XII) chain precursor	IPI00189714	26/15
Col6a2	Similar to procollagen, type VI, alpha 2	IPI00372839	24/1
Col6a1	Predicted similar to collagen alpha-1(VI) chain precursor	IPI00371853	34/10
